# Comparison of the double loop knot stitch and Kessler stitch for Achilles tendon repair: A biomechanical cadaver study

**DOI:** 10.1371/journal.pone.0243306

**Published:** 2020-12-03

**Authors:** Stephan Frosch, Gottfried Buchhorn, Thelonius Hawellek, Tim Alexander Walde, Wolfgang Lehmann, Jan Hubert

**Affiliations:** 1 Department of Trauma Surgery, Orthopaedics and Plastic Surgery, University Medical Center Goettingen, Göttingen, Germany; 2 Department of Orthopaedics, University Medical Center Hamburg-Eppendorf, Hamburg, Germany; Universidade Federal Fluminense, BRAZIL

## Abstract

Tendon elongation after Achilles tendon (AT) repair is associated with the clinical outcome. Reliable suture techniques are essential to reduce gap formations and to allow early mobilization. Cyclic loading conditions represent the repetitive loading in rehabilitation. The aim of this study was to compare the Kessler stitch and double loop knot stitch (DLKS) in a cyclic loading program focussing on gap formation. Sixteen human cadaveric ATs were transected and sutured using either the Kessler stitch or DLKS (eight matched pairs). The suture-tendon configurations were subjected to cyclic loading and additional ultimate load to failure testing using the Zwick 1446 universal testing machine. Each AT survived cyclic loading, with a mean gap formation less than 5 mm after 1000 cycles. The mechanical properties of the Kessler stitch and DLKS were not significantly different after cyclic loading with a mean displacement of 4.57 mm (± 1.16) for the Kessler stitch and 4.85 mm (± 1.14) for the DLKS (P = .76). There were no significant differences in the ultimate load testing (P = .85). Both bioprotective techniques prevent excessive gaping in cyclic testing when tendon loading is moderate. Our data and those from literature of gap formation in cyclic and ultimate loading allow the conclusion, that early aggressive AT loading after repair (e.g. full weightbearing) overstrain simple as well as complex suture configurations. Initial intraoperative tightening of the knots (preloading) before locking is important to decrease postoperative elongation.

## Introduction

The Achilles tendon (AT) is the most frequently ruptured tendon, with increasing incidence [1, 2]. AT rupture commonly occurs during sports activities in males during their third or fourth decades (i.e., “weekend warriors”) [2, 3]. After AT rupture, patients often do not recover full strength and function, even after extended rehabilitation [4–6]. Although strength and functional outcome are key issues in treatment, the re-rupture rate is commonly the main variable for assessing the success of treatment.

Recent literature reports an equal re-rupture rate after operative and non-operative treatment when performing early mobilization in rehabilitation [7, 8]. However, operative treatment could be beneficial for younger active patients and athletes, as the functional outcome in terms of athletic abilities (jump test, hopping test, heel-rise endurance test) is better in the operative group [7].

Tendon elongation is commonly observed after operative and non-operative procedures for AT rupture [9–11]. Tendon elongation correlates with the clinical outcome and causes morbidity and strength deficits of 10% to 30% after AT rupture [5, 9]. Accordingly, patients with less elongation have better clinical outcomes [9, 12]. Excessive gap formation in the early postoperative stage due to failure of the suture-tendon complex has been attributed to tendon elongation [13, 14]. Therefore, considering an equal re-rupture rate compared to non-surgery and the risk of significant operative complications (up to 27%), a reliable suture technique preventing excessive gap formation is needed [15].

The double loop knot stitch (DLKS) has superior biomechanical properties in rotator cuff repair [16]. The self-tightening property of the loop knot enhances tissue grip as axial strain increases, and its transverse compression allows more effective grasping of frayed tendon tissue. Furthermore, the loop characteristic enables the surgeon to only grab a small part of each side of the tendon compared to suture techniques transversely compressing the entire diameter of the tendon, which may decrease microcirculation [17]. The Kessler stitch is one of the most commonly used techniques and a standard configuration in AT repair [18–20].

The aim of this study was to analyse the biomechanical properties of DLKS compared to the Kessler stitch with a focus on the resistance of suture tendon slippage (gap formation) under cyclic loading conditions and ultimate load testing.

## Materials and methods

### Sample preparation

Sixteen (eight matched pairs) human cadaveric ATs including the triceps surae muscle and the calcaneus were harvested from the bodies of three males (37.5%) and five females (62.5%) with a mean age of 89.75 (±5.75) years. Approval was obtained from the Ethics Commission of the University Medical Center Göttingen (Protocol Number: 24/7/13). All of the harvested specimens were intact without past injuries in medical history. An additional visual and palpatory examination of the Achilles tendon was performed to confirm the integrity of the tendon and to rule out possible damage to the tendons. The specimens were stored at -38°C and thawed at room temperature 12 hours before the experiment. The ATs were transversely dissected 45 mm proximal to the tuber calcanei using a scalpel to simulate a rupture.

### Suture

**Fig 1:**
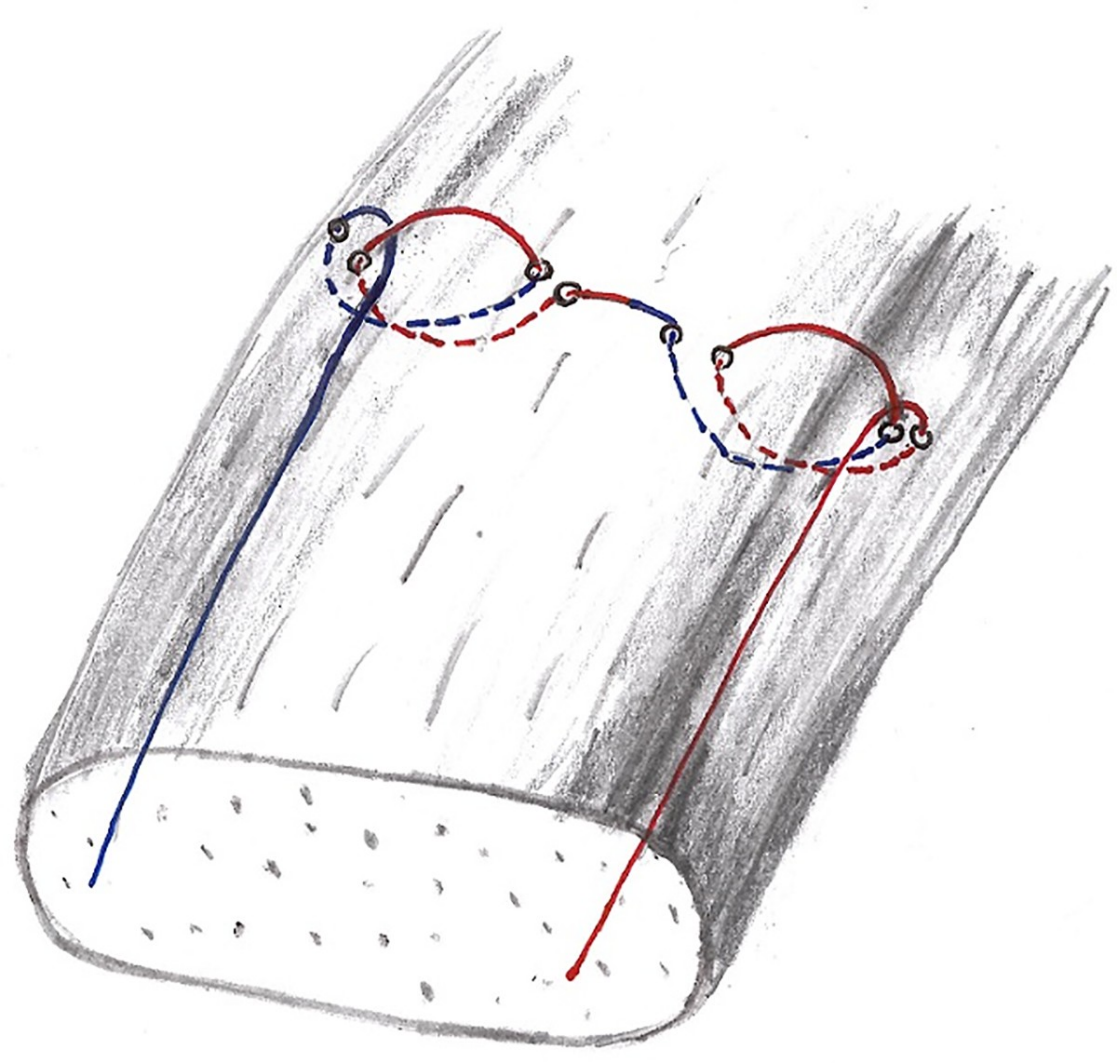
Double Loop Knot Stitch (DLKS).

In preliminary tests with two human cadaveric AT, we first used a Fiberwire thread to eliminate the factor “thread tear force” and only evaluate the Kessler and DLKS knot strength. In comparison with the PDS thread, it turned out that the thread tear force was not the limiting factor in the test process (no breakage of the PDS thread was observed). According to the clinical application, we decided to use a PDS thread. The suture material was an absorbable polydioxanone 2–0 thread (PDS–Ethicon, Norderstedt, Germany). The tendons were sutured 2 cm proximal and 2 cm distal to the transection plane with a round needle. Care was taken to embrace comparable portions of the tendons. Each matched paired AT was allocated at random to two groups, performing either the Kessler suture technique or the DLKS technique (Fig 1) [16, 21, 22]. The randomization was carried out by rolling the dice (numbers 1 to 6) with a fixed paired assignment of the numbers to a suture technique on the right AT and the corresponding other suture technique on the left AT of a corpse.

Each of the two knots of the DLKS requires two horizontal passes through the tissue to form a sling with a knot that tightens as tension on the thread increases (Fig 1). In AT repair, two mirrored DLKS (one on each side of the rupture) are applied. Seven ties were used to secure the DLKS and Kessler stitches. All sutures were performed by the First Author (SF) who is a senior orthopedic surgeon.

### Biomechanical testing

**Fig 2:**
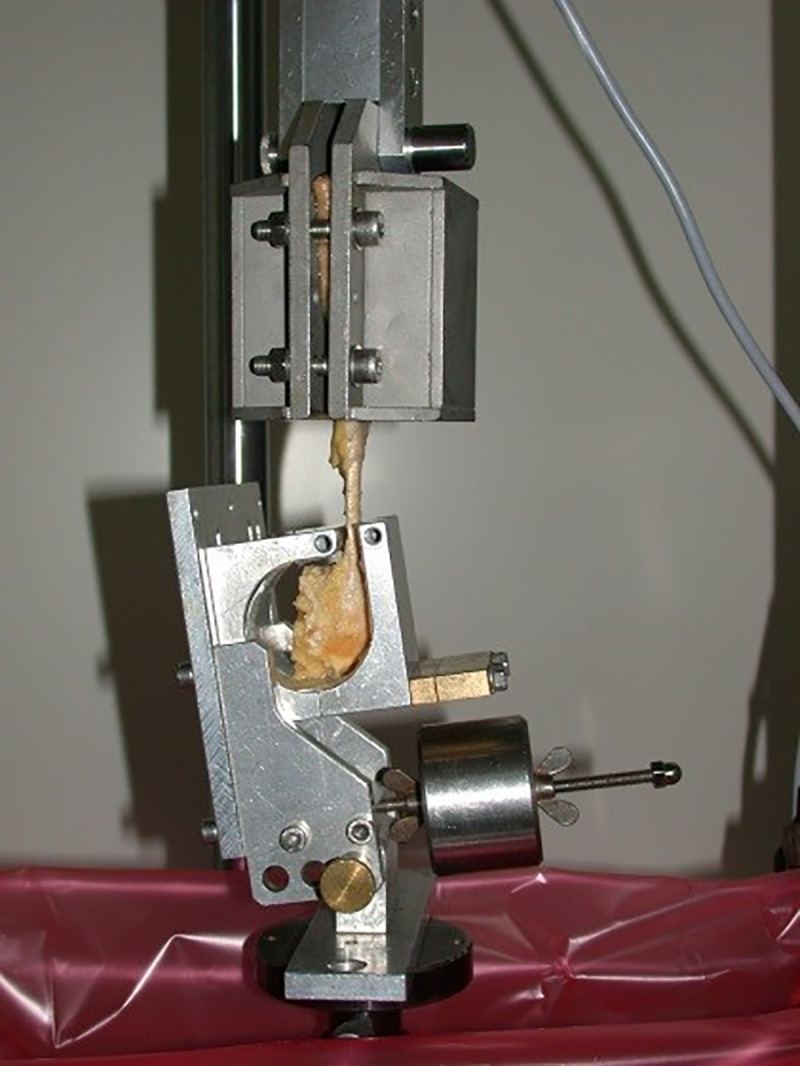
Setup of the Zwick testing machine. The muscle is clamped between metal brackets filled with dry ice. The calcaneus on the button will be cemented after proper positioning.

**Fig 3:**
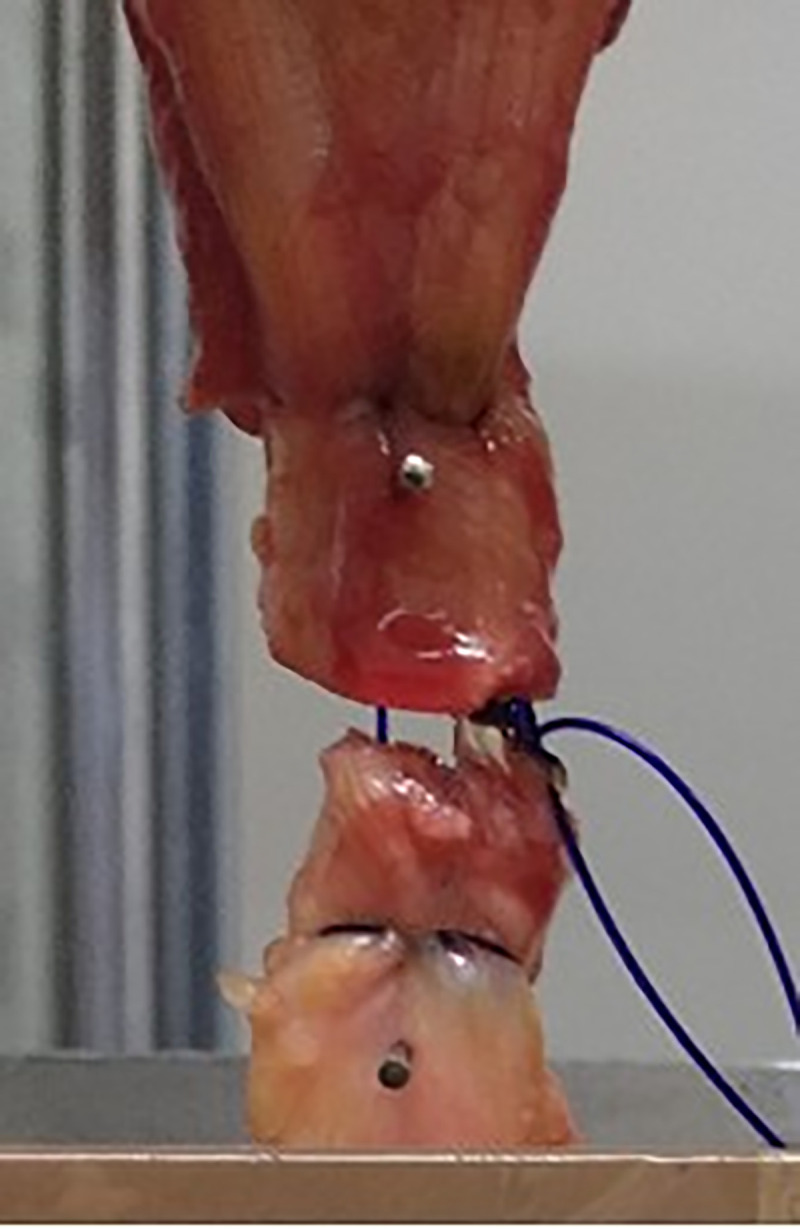
DLKS repair after 500 cycles. The two-sided branches of the DLKS are seen at the distal end.

**Fig 4:**
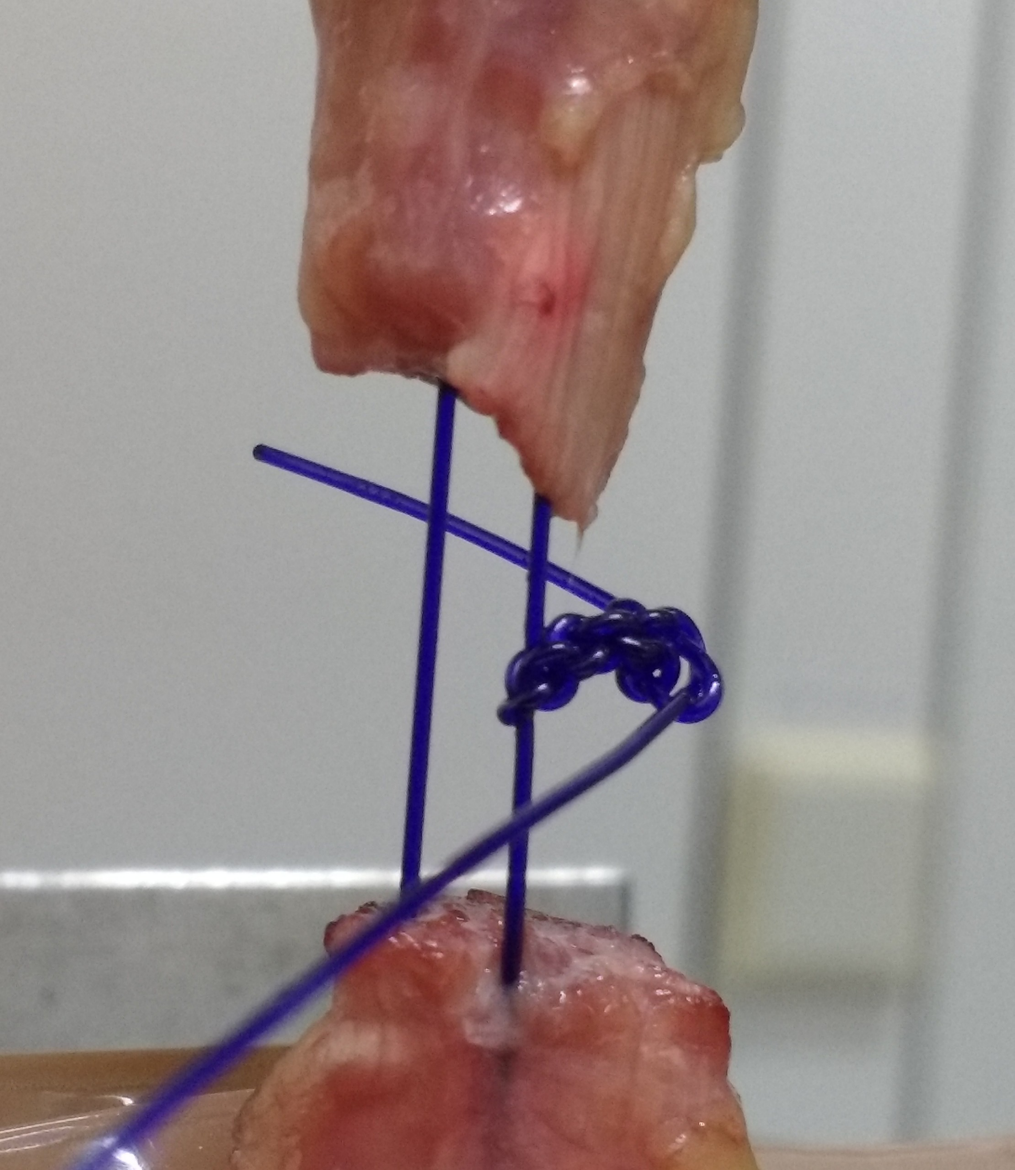
Kessler stitch during ultimate load to failure testing.

The samples were subjected to unidirectional, cyclic loading and subsequent ultimate load to failure using a Zwick 1446 universal testing machine (UTM; Zwick-Roell AG, Ulm, Germany). The muscle was clamped under compression by two metal brackets [23]. To prevent slippage, metal bags surrounding the brackets were filled with pellets of dry ice, freezing the clamped part of the muscle. The downwards protruding muscle and tendon remained unaffected. The calcanei were fixed and cemented in a custom-made fixing box attached to the base of the Zwick machine (Fig 2). The data were recorded using testing software (textXpert V 112.1, Zwick-Roell AG, Ulm, Germany). After pre-tension to 20 N, the samples were stressed axially at a displacement rate of 20 mm/s. A total of 1000 cycles were performed and the samples loaded from 5 to 20 N in each cycle (Fig 3). The displacement rate and the cyclic loading regime are based on previous studies and appear to be in physiological range [14, 19, 24–28]. The displacement was recorded at 100, 500, 750, and 1000 cycles. Biomechanical failure in ultimate load testing was defined as an 80% loss of the ultimate tensile strength independent of the failure mode (Fig 4) [16, 29].

### Statistical analysis

The quantile-quantile plot revealed a normal distribution of the data. Mean +/- standard deviation; median (min, max) was stated for continuous variables and absolute as well as relative frequency was given for categorical ones. Statistical analyses were performed using either linear mixed effects models or paired t-test.

The significance level was set to alpha = 5% for all statistical tests. In case of multiple testing situation, raw p-values were adjusted by the method of Bonferroni-Holm. All analyses were performed with the statistic software R (version 3.1.2, www.r-project.org) using the R-package lme4 for the linear mixed effects model. Based on the literature, we chose a sample size of N = 8 for each group [14, 30–35].

## Results

All suture-tendon configurations survived the cyclic loading procedure and were subsequently loaded to failure. Displacements (gap formation) were not significantly different after cyclic loading between the Kessler and DLKS groups (P = .76; Table 1). In both groups, gap formations did not exceed 5 mm.

**Table 1:** Mean displacement during cyclic loading (standard deviation) [minimum, maximum].

	Gap after 100 cycles (mm)	Gap after 500 cycles (mm)	Gap after 750 cycles (mm)	Gap after 1000 cycles (mm)	P value after 1000 cycles
DLKS	1.57 (± 0.52) [0.51, 2.29]	3.38 (± 0.9) [1.76, 4.7]	4.12 (± 0.91) [3.02, 5.57]	4.85 (± 1.16) [4.11, 6.91]	0.76
Kessler	1.13 (± 0.46) [0.62, 1.92]	2.97 (± 0.8) [1.98, 4.42]	3.89 (± 0.97) [2.69, 5.26]	4.57 (± 1.14) [3.04, 6.68]	

**Fig 5:**
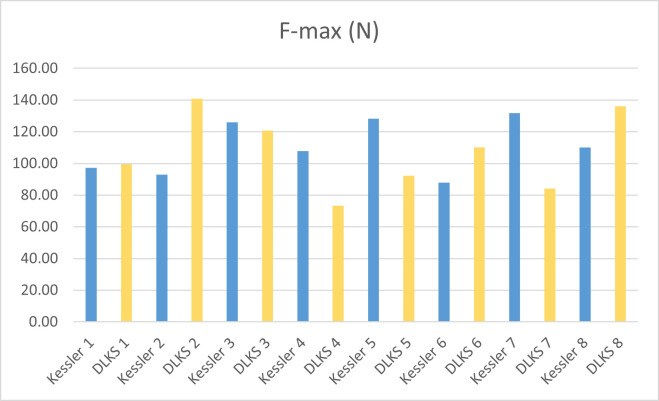
Single values from ultimate load to failure testing.

The ultimate load to failure testing yielded 110.27 N (± 16.96) in the Kessler group and 107.15 N (± 24.28) in the DLKS group (P = .85; Table 2 and Fig 5).

**Table 2:** Results of the ultimate load to failure testing (standard deviation) [minimum, maximum].

	Force-max (N)	P value (suture technique)
DLKS	107.15 (± 24.28) [73.34, 140.95]	0.85
Kessler	110.27 (± 16.96) [87.92, 131.89]	

In the Kessler group, the failure modes at ultimate loading were suture rupture (three times) and pull out of the suture (five times). In the DLKS group, the failure modes were suture rupture (five times) and pull out (three times). Donor sex (P = .96) and age (P = .47) had no significant effect on the dependent variables.

## Discussion

Both the DLKS and the Kessler stitch showed good and comparable biomechanical properties in cyclic loading (5–20 N), each with a mean gap formation of less than 5 mm after 1000 cycles. There were no significant differences in cyclic loading (P = .76) and in the ultimate load testing (P = .85) between both suturing techniques.

Mechanical stimulation of the AT tendon after repair promotes tendon healing and improves the postoperative outcome [10, 12]. In this context, early mobilisation of the ankle reduces tendon elongation, as well as the re-rupture rate, and improves tendon healing and functional outcome [9, 10]. However, the degree of tendon elongation after AT repair and side-to-side differences in heel-rise height cause weakness in the end-range plantar flexion of the ankle [5, 12, 14, 36]. Therefore, experimental models are necessary to gain experience with adequate early mechanical loading (cyclic loading) and weightbearing (ultimate load).

Cyclic loading, rather than ultimate load to failure configurations, simulates repetitive loading of the tendon during the early stages of rehabilitation. Gap formation exceeding 5 mm is considered a clinically relevant failure of the suture-tendon complex [14, 36]. In a human cadaver study, Clanton et al. investigated a modified Kessler stitch and three percutaneous repair techniques using a cyclic loading protocol [37]. After 250 cycles with a loading range of 20–100 N, the modified Kessler stitch had a mean gap formation of 5.2 mm after the first 250 cycles, whereas the resulting gaps for percutaneous stitches were significantly wider (Achillon stitch 9.9 mm, PARS stitch 12.2 mm, SpeedBridge 10.0 mm). Considering the modification to the Kessler stitch technique (three solitary Kessler stitches) and the lower cycle range (250 vs. 1000), the data support our findings. We demonstrated gap formation less than 5 mm after cyclic loading for both stitches, with the same biomechanical properties for both stitching techniques. Our cyclic loading protocol is similar to the protocol of Herbort et al. [18]. They demonstrated gap formation of 5.58 mm for the Kessler stitch after 1000 cycles (5–20 N), representing comparable results to our findings. The somewhat smaller gap formation with the Kessler stitch (4.57 mm) is presumably due to the pre-tensioning of our suture-tendon complex.

In ultimate load to failure tests of human cadaver AT repairs, previous experimental studies have focused primarily on the maximum load value rather than gap formation to evaluate the efficiency of suture configurations.Sadoghi et al. included 11 studies in a systematic review, reporting of a mean 222.7 N for the initial strength of 196 cadaver AT repairs [38]. Eight of the eleven studies did not report gap formation [18, 19, 32, 34, 39–41]. Therefore, the integrity of the suture configuration in terms of possible excessive gapping during the tendon loading remains unclear. The few reported load-displacement graphs in these studies demonstrate gap formations between 3 and 6 cm, representing clinical failure [18, 40]. Two studies, Labib et al. and Shepard et al., defined 10 mm gap formation as failure of the suture and reported a failure force of 40 N and 81 N for the complex Krackow stitch technique [42, 43]. Their results are consistent with our findings and show that suture configurations in AT repair may not sustain aggressive tendon loading considering the estimated force of 370 N on the AT when walking with an ankle immobiliser in neutral and 191 N with 1 inch heel lift [44]. Consequently, early full weightbearing after AT repair may lead to suture failure due to excessive gapping and subsequent tendon lengthening [5, 45].

To improve the holding strength of simple suture techniques, such as the Kessler stitch and DLKS, additional suture modification like epitendinous sutures and plantaris tendon augmentation are needed [14, 19]. Furthermore, increasing the core strands for double or triple application of a simple stitch, such as the Kessler suture, creates a significantly stronger suture-tendon construct [34, 46]. In our daily routine, we prefer using several simple stitches rather than a complex, tendon-constricting suture technique, such as the Krackow, Giftbox, or triple bundle technique in order to preserve microvascularisation of the tendon [20, 32]. Praxitelous and co-workers pointed out that higher maximum blood flow to the AT after repair significantly correlates with improved patient-reported and functional outcomes [47]. Furthermore, Kraemer et al. demonstrated a decrease in capillary perfusion and venous stasis after AT suture in an animal model using a simple Kirchmeyer suture [48]. Complex, circular-constricting sutures with multiple strands strangulate the tendon and reduce microvascularisation compared to the simple DLKS and Kessler stitch, which are bioprotective techniques [32].

The superior biomechanical properties of the DLKS in rotator cuff repair could not be shown in AT repair when compared to a gold standard stitch. In progressive cyclic loading the cinching loop tightens up to a certain extent, which increases the thread-length in between the knots and therefore slightly increasing elongation [49]. This effect seems to add up as the two consecutive DLKS in AT repair have 4 self-cinching loop knots. Therefore, initial intraoperative tightening of the knots (preloading) before locking the stitch is important to decrease postoperative elongation. It is possible to place two separate DLKS at the proximal and distal of the rupture and tie the opposing threads on each side of the tendon (two knots). Alternatively, both DLKS can be placed consecutively with one continuous thread and one final locking knot. It should be noted that tightening of two consecutive DLKS with one thread is more difficult because of the self-cinching mechanism. In cases of highly frayed tendon stumps, the DLKS may be favourable due to the self-cinching properties of the knot, which allow smaller parts of the frayed tendon to be grasped.

A limitation of this study is the mean age of the specimens, which was 89 years, whereas the typical AT rupture occurs in the fourth decade of life. The difference in tissue quality may explain the results, and one could assume that with specimens from younger donors, the gap formation would be even smaller. Imaging (e.g. ultrasound) of the tendons to exclude intratendinous damage would have reduced potential bias. Furthermore, clean transverse transections of a tendon with different preconditions than a ruptured AT with fraying stumps could alter suture difficulty.

In conclusion, the DLKS is a reliable alternative to the Kessler stitch with similar mechanical properties in this cadaveric study. Both techniques protect the suture-tendon configuration when cyclic tendon loading is moderate.

## Supporting information

S1 Data (DOCX)
