# Supplementary material for: Comparison of the double loop knot stitch and Kessler stitch for Achilles tendon repair: A biomechanical cadaver study
Source: PLoS One. 2020 Dec 3;15(12):e0243306. doi: 10.1371/journal.pone.0243306 (PMC7714161; doi:10.1371/journal.pone.0243306)
Supplement: S1 Data — (DOCX) [file pone.0243306.s001.docx]

|  | 2101-ri | 2101-le | 2102-ri | 2102-le | 2103-ri | 2103-le | 2104-ri | 2104-le |
| --- | --- | --- | --- | --- | --- | --- | --- | --- |
| Speci-mens | 160810 DL | 160810 K | 160811 DL | 160811 K | 160929 K | 160929 DL | 161006 K | 161006 DL |
| F max (N) | 99.39 | 97.19 | 140.95 | 92.98 | 126.02 | 120.63 | 107.77 | 73.34 |
| cycles (in mm) | |  |  |  |  |  |  |  |
| 0 – 100 | 1.88 | 0.62 | 1.34 | 1.62 | 0.79 | 0.51 | 1.03 | 1.83 |
| 101 – 500 | 4.7 | 2.64 | 2.78 | 4.42 | 3.67 | 1.76 | 2.1 | 3.82 |
| 501 – 750 | 5.18 | 2.85 | 3.02 | 4.69 | 4.93 | 3.02 | 2.69 | 4.4 |
| 751 – 1000 | 6.91 | 3.73 | 4.14 | 4.81 | 5.62 | 4.17 | 3.04 | 4.67 |

Raw data of cyclic loading (mm) and ultimate load test (F max – in N). ri = right Achilles tendon (AT). Le = left AT tendon. DL = DLKS. K = Kessler stitch.

|  | 2105-ri | 2105-le | 2106-ri | 2106-le | 2107-ri | 2107-le | 2108-ri | 2108-le |
| --- | --- | --- | --- | --- | --- | --- | --- | --- |
| Speci-mens | 161013 DL | 161013 K | 161020 K | 161020 DL | 161026 DL | 161026 K | 160810 DL | 160810 K |
| F max (N) | 92.29 | 128.19 | 87.92 | 110.22 | 84.18 | 131.89 | 136.17 | 110.20 |
| cycles (in mm) | |  |  |  |  |  |  |  |
| 0 – 100 | 1.45 | 0.79 | 1.92 | 2.29 | 1.66 | 0.7 | 1.62 | 1.07 |
| 101 – 500 | 2.84 | 3.21 | 2.83 | 3.44 | 3.91 | 2.89 | 3.75 | 1.98 |
| 501 – 750 | 3.91 | 5.26 | 3.87 | 3.86 | 5.57 | 3.49 | 4.01 | 3.32 |
| 751 – 1000 | 4.17 | 6.68 | 4.36 | 4.11 | 6.48 | 4.08 | 4.18 | 4.27 |

Raw data of cyclic loading (mm) and ultimate load test (F max – in N). ri = right Achilles tendon (AT). Le = left AT tendon. DL = DLKS. K = Kessler stitch.
